# Introduction to the special collection: developing valid psychological measures for populations impacted by humanitarian disasters

**DOI:** 10.1186/s13031-020-00260-6

**Published:** 2020-02-21

**Authors:** Andrew Rasmussen, Nuwan Jayawickreme

**Affiliations:** 1 Psychology, Fordham University, 441 E Fordham Rd, Dealy Hall 236, Bronx, NY USA; 2 Psychology, Manhattan College, Bronx, NY USA

Individuals displaced due to humanitarian disasters—currently 70.8 million [1]—suffer from high rates of mental health problems. Although rates of depression and posttraumatic stress disorder (PTSD) in these populations range widely [2, 3], it is estimated that at least a quarter of all survivors suffer from either depression or PTSD [4]. In order to correctly identify those displaced individuals who are experiencing clinically significant mental distress, it is imperative that psychosocial professionals have measurement tools that are valid and reliable for the populations with which they are working.

In the early years of humanitarian psychosocial interventions (i.e., the 1980s and 1990s), it was common practice when assessing mental health in disaster- and conflict-affected populations to limit psychometric assessment of measurement tools to simply reporting Cronbach’s alpha—a necessary but hardly sufficient practice when working with novel populations. If an adequate alpha were reported, the validity and reliability of the measure were typically assumed across populations. A few measurement tools were developed and validated for specific forcedly displaced populations (e.g., the Harvard Trauma Questionnaire for Cambodian, Laotian, and Vietnamese refugees [5]); however, this led to other researchers using these measures and claiming that they had been validated for refugee populations in general. In a critical review of 183 studies almost 20 years ago, Hollifield and colleagues [6] found that the majority of research articles published on the mental health of refugee and related populations to that point included data from instruments whose scores had limited or untested validity and reliability in refugee populations. Furthermore, half reviewed did not report any statistical properties of the instruments used, and only 12 articles used instruments that had been developed specifically for displaced populations. In a commentary 5 years later Bass, Bolton, and Murray [7] noted that there had been few attempts at that time to develop instruments whose scores were valid and reliable for use in communities affected by humanitarian disaster. Thankfully, in the past decade, there has been a growing realization among those working in psychosocial intervention that the reliability and validity of measurement tools used in populations other than those the measures were originally developed for must be demonstrated, not simply assumed (e.g., [8, 9]).

Researchers and practitioners who focus on populations impacted by humanitarian disasters and civil conflict have started to pay more attention to the psychometric properties of the measures they use (e.g., [10, 11]). This has led to a burgeoning area of study that spans the literatures of global mental health, transcultural psychiatry, refugee trauma and psychometrics. By proposing a special collection of papers in *Conflict and Health* that focuses on the psychometrics of commonly used measurement tools in disaster contexts, we hoped to provide the field in general with an opportunity to reflect on current approaches to developing valid measures for use in disaster contexts, and a touchstone for moving forward. We aimed to showcase state-of-the-art methodologies that are being used to support the use of such tools in humanitarian disaster settings. We hope that readers of this special collection will be able to identify the appropriate methodological approaches that suit their needs, and have a clear sense of the specific steps they need to take to support the use of a measure for the specific population in which they are working.

The six papers contained within this special collection span a wide range of populations impacted by humanitarian disasters, a wide range of tools measuring a variety of psychological constructs, and a wide range of psychometric approaches used to evaluate the use of these tools within these populations. Samples include young adult secondary school students in Haiti [12], internally displaced adults and veterans from Ukraine [13], Eritrean refugees living in Ethiopia [14], refugees from the Darfur region of Sudan living in Chad [15], refugees from Iraq, Iran, Sri Lanka and other countries currently residing in Australia [16] and a national, representative sample of Sri Lankan adults affected by civil war [17]. Psychological constructs measured include the psychopathological constructs most common to the psychosocial aid literature, posttraumatic stress disorder (PTSD), major depression, and anxiety [12, 16, 17], but also substance abuse [13], cultural concepts of distress [15] and broader phenomena related to wellbeing [14]. Psychometric approaches include confirmatory factor analysis (CFA) [14, 16, 17], receiver operating characteristics (ROC) [12], item response theory (IRT) [13], and dynamic network analysis [15]; one study even shows how qualitative methods can be integrated to develop and validate tools [13].

The articles in this special collection address a series of key issues faced by practitioners attempting to measure mental health constructs accurately and identify those in need of intervention. They are:

1. *Efficiently identify salient problems in populations impacted by humanitarian disasters, and create valid, reliable and concise measures of mental distress.* In many cases, psychosocial practitioners entering a new post-disaster context have to quickly assess the salient psychosocial problems facing those populations and develop accurate assessments of those problems that are brief and not too burdensome on respondents. Doty and colleagues [13] provided an impressive showcase of how to efficiently engage in this practice. They first conducted quick ethnography [17] to gain information about salient mental health problems and indicators of functional impairment in internally displaced individuals and veterans in Ukraine. Based on that information they selected existing instruments depression, PTSD, anxiety, and substance use and modified them using the information that they gleaned from their qualitative studies. Following this, they used IRT to shorten their instruments. The result was a comprehensive measure of psychosocial problems, valid and reliable for displaced and veteran Ukrainians, easy to use, and not too burdensome on respondents due to its conciseness.
2. *Establish measurement invariance of translated measures.* Displaced and conflict-affected populations are almost invariably from cultures outside of those from which common measures of psychological distress have been developed. If practitioners wish to compare scores from these measurement tools between individuals or samples from different cultural groups, they must be confident that the tools are measuring the construct of interest in the same way. In other words, they must establish that these measurement tools have measurement invariance (also referred to as measurement equivalence). Tay and colleagues [17] demonstrate the use of a multigroup CFA-based statistical method designed for diverse populations—the alignment method—to establish measurement invariance of a commonly used measure of depression and anxiety, the Hopkins Symptoms Checklist, in an ethnically- and geographically-diverse Sri Lankan sample.
3. *Test the validity of new models of established diagnostic constructs.* PTSD has had its diagnostic criteria revised multiple times since it was introduced into the third edition of the Diagnostic and Statistical Manual of Mental Disorders (DSM-III) [18]. The most recent edition of the DSM (DSM-5) [19] made significant changes to the PTSD diagnosis, increasing the number of symptoms from 17 to 20 and increasing the number of symptom clusters from three to four. Using CFA, Specker and colleagues [16] evaluated the construct validity of a self-report measure of the newly revised PTSD model in a sample of adult refugees living in Australia. They found that a 6-factor *Anhedonia* model, comprising the symptom clusters of re-experiencing, avoidance, negative affect, anhedonia, dysphoric arousal and anxious arousal, fit the data better than the 4-factor DSM-5 model, replicating a number of studies published since the advent of DSM-5 (for a review, see [20].
4. *Identify scale scores that identify clinically significant distress.* Total score “cut-offs” developed for one particular population may not be indicative of clinically significant mental distress in another population. This can be the case even for a measure that has been developed for a closely related population, using the same culturally contextualized idioms of distress. Legha and colleagues [12] used ROC curve analyses to identify the clinically significant cut-off score for the Zanmi Lasante Depression Symptom Inventory (ZLDSI) in Haitian young adults in secondary school and found that a slightly different score identified individuals with depression than had been found in the original ZLDSI development study. Without such findings it is possible that the community health workers working with this population would have missed individuals with clinically significant distress, a classic problem of false negatives.
5. *Establish validity of measures of general well being developed in North America and Europe.* More and more psychologists are examining the role that psychological constructs related to resilience play in determining the mental health of disaster-impacted populations (e.g., [21, 22]). Getnet and Alem [14] assessed the validity and reliability of a scale measuring such a psychological construct, namely sense of coherence, in adult Eritrean refugees living in Ethiopia. Using CFA, they identified four items that did not work well in this population; however, a shortened version of the scale fit well, and demonstrated convergent validity with measures of social support and coping.
6. *Use of novel statistical techniques to identify individuals with clinically significant mental distress.* Although the research literature generally focuses on identifying patterns of relationships between reflective indicator measures of mental health and functional impairment across studies, the non-latent assumptions of network analysis may be better suited to the practical needs of specific settings. Using archival data of Darfur refugees living in Chad Mootoo and colleagues [15] demonstrated how dynamic network analysis can be used to identify relationships between specific daily stressors, experienced traumas, indicators of functional impairment and symptoms of *hozun*, a local depression-like construct, and proposed that such analyses might be particularly useful in formative evaluations. Mootoo and colleagues [15] compared their findings to past analyses of the same data, and found that network analysis provided findings that were more specific to the setting in which data were collected.

It is our hope that these papers will provide researchers and practitioners of psychosocial humanitarian aid with a sense of the state-of-the-art psychometric tools at their disposal. Although in no way exhaustive, we feel that this collection presents a useful introduction to the range of approaches available for who need to measure mental health in disaster- and conflict- affected populations.
